# Assessing Neutralized Nicotine Distribution Using Mice Vaccinated with the Mucosal Conjugate Nicotine Vaccine

**DOI:** 10.3390/vaccines9020118

**Published:** 2021-02-03

**Authors:** Nya L. Fraleigh, Jordan D. Lewicky, Alexandrine L. Martel, Francisco Diaz-Mitoma, Hoang-Thanh Le

**Affiliations:** 1 Department of Research, Health Sciences North Research Institute (HSNRI), 56 Walford Road, Sudbury, ON P3E 2H3, Canada; nfraleigh@hsnri.ca (N.L.F.); jlewicky@hsnri.ca (J.D.L.); amartel@hsnri.ca (A.L.M.); 2 Department of Chemistry & Biochemistry, Laurentian University, 935 Ramsey Lake Road, Sudbury, ON P3E 2C6, Canada; fdiazmitoma@vbivaccines.com; 3 VBI Vaccines, Inc., 310 Hunt Club Road Suite 201, Ottawa, ON K1V 1C1, Canada; 4 Medical Sciences Division, Northern Ontario School of Medicine, 935 Ramsey Lake Road, Sudbury, ON P3E 2C6, Canada

**Keywords:** nicotine vaccine, mucosal, intranasal, nicotine distribution, nicotine analysis method

## Abstract

Tobacco smoking continues to be a global epidemic and the leading preventable cause of cancer and cardiovascular disease. Nicotine vaccines have been investigated as an alternative to currently available smoking cessation strategies as a means to increase rates of success and long-term abstinence. Recently, we demonstrated that a mucosal nicotine vaccine was able to induce robust mucosal and systemic antibodies when delivered heterologously using intranasal and intramuscular routes. Herein, we investigated the neutralization ability of the anti-nicotine antibodies using both intranasal and intracardiac nicotine challenges. Combining the extraction of lyophilized organ samples with RP-HPLC methods, we were able to recover between 47% and 56% of the nicotine administered from the blood, brain, heart, and lungs up to 10 min after challenge, suggesting that the interaction of the antibodies with nicotine forms a stable complex independently of the route of vaccination or challenge. Although both challenge routes can be used for assessing systemic antibodies, only the intranasal administration of nicotine, which is more physiologically similar to the inhalation of nicotine, permitted the crucial interaction of nicotine with the mucosal antibodies generated using the heterologous vaccination route. Notably, these results were obtained 6 months after the final vaccination, demonstrating stable mucosal and systemic antibody responses.

## 1. Introduction

Despite the risks associated with smoking tobacco, it continues to be an ongoing global public health pandemic with approximately 1.3 billion people who smoke tobacco [1]. Smoking tobacco not only increases risks associated with cancer, respiratory and cardiovascular diseases but results in a substantial social and economic burden that in Canada is responsible for 18% of deaths and over CAD 16 billion of direct and indirect costs [2].

Smoking cessation aides, such as nicotine replacement products and pharmacotherapeutics, are readily available to help those who wish to quit smoking, however, success rates remain low with a high risk of relapse [3]. Nicotine vaccines have long been considered the next step as a therapeutic for smoking cessation because of the lack of side-effects [4] and risk for abuse [5], and the potential to increase abstinence rates. Additionally, anti-nicotine antibodies would have a longer half-life, as compared to other pharmacotherapeutics and nicotine itself, resulting in dose sparing [5] that inevitability increases the safety profile of the vaccine. Over the last 20 years, several nicotine vaccines have successfully advanced to various stages of clinical trials, but were unable to successfully obtain significant abstinence rates between experimental groups [6,7,8,9,10]. The premise behind the development of these vaccines is that the nicotine hapten is small and not immunogenic enough to be recognized as an antigen by the immune system, but with an effective adjuvant, the immune system can direct an immune response resulting in nicotine-specific antibodies. However, these vaccines continue to rely on parenteral administration, limiting the availability of the anti-nicotine antibodies to the systemic circulation, which alone may be unable to efficiently capture nicotine.

We have previously demonstrated that utilizing a heterologously (intramuscular (IM)/intranasal (IN)) administered, mucosally directed nicotine vaccine induced robust mucosal and systemic antibodies directed towards nicotine, resulting in two levels of protection [11]. Previously, we had demonstrated the ability of the IN administered mucosal conjugate nicotine vaccine to neutralize nicotine when mice were challenged with [^3^H]-nicotine [12]. While effective, recently there has been a shift away from using [^3^H]-nicotine for nicotine challenges, and towards using nicotine tartrate to assess the distribution of nicotine by GC/MS, LC/MS or HPLC. However, unlike [^3^H]-nicotine, that is typically if not always delivered intravenously (IV), non-tritiated forms of nicotine have been delivered subcutaneously (SC) [13,14,15,16], IV [17], or intraperitoneally (IP) [18], all of which resulted in low levels of recovery, regardless of the dose administered. This does not necessarily permit nicotine vaccines evaluated in vivo to be properly assessed due to the low recovery rates in the brain and the sera.

Herein, we demonstrate the efficacy of the antibodies by evaluating the neutralizing ability of the antibodies generated by the bacterial derived adjuvant (BDA)-conjugate nicotine vaccine in vivo administered either IM/IN or IM, using either an intranasal (IN) instillation or an intracardiac (IC) injection of 0.09 mg/kg of pure nicotine (equivalent to 0.257 mg/kg nicotine tartrate); representing both a mucosal and systemic administration of nicotine, respectively. The stability of the antibodies was determined by conducting the challenge 6 months after the final vaccination and assessing the ability of the complex to be stable for the duration of a 10 min challenge protocol. For each of the vaccination and challenge routes, the recovery range using the combined extraction of lyophilized organs and RP-HPLC was greater than 47% for the four organs/fluids collected: brain, lung, heart, and blood. When nicotine was delivered mucosally, the IM/IN vaccinated mice, with antibodies in both the lung and the sera, were the most effective at neutralizing nicotine with significantly increased levels detected in the lung as compared to the nonvaccinated mice and a 3.54-fold (72%) decrease of nicotine in the brain.

## 2. Materials and Methods

### 2.1. Animals

Female 6–8 week old BALB/c mice were purchased from Charles River (St. Constant, QC, Canada) and housed at the animal care facility at Laurentian University. Mice were housed as previously reported [11], randomly placed in groups of five, and were allowed to acclimatize to their surroundings for at least 1 week prior to the start of the vaccination protocol. All protocols were approved by the Animal Care Committee at Laurentian University and the Biohazard and Biosafety Committee at HSNRI.

### 2.2. Vaccine and Vaccination Protocol

The bacterial-derived adjuvant (BDA)-conjugate nicotine vaccine was prepared in house (HSNRI, Sudbury, ON, Canada) as previously reported [11,12,19,20]. Mice were vaccinated under light anesthesia using isoflurane once every 3 weeks using either the homologous (3 IM) or heterologous strategy (1 IM simultaneously with an IN and 2 subsequent IN) with either PBS or the nicotine vaccine as previously described [12]. Sera was collected 2 weeks after the final vaccination (56 day time point) using the saphenous vein and stored at −20 °C until ELISA analysis.

### 2.3. Clinical Observations

Mice were observed daily and monitored for signs of distress or vaccine associated toxicity which included changes in gait, posture, and behavior. As part of basic toxicity associated measurements, the mice were weighed weekly starting from day 0 (first vaccination event) for 9 weeks. Corporal temperatures of the mice were also recorded before and 24 h after each vaccination event to monitor changes in temperature that would correlate with a fever.

### 2.4. Nicotine Challenge

Mice were bled and weighed prior to the preparation of nicotine for the nicotine challenge. Briefly, nicotine hydrogen tartrate (Sigma Aldrich, St. Louis, MO, USA) was dissolved in formic acid acidified water (0.1% *v*/*v*, LC/MS Grade, Fisher Scientific) at a concentration of 2–8 mM, depending on the challenge route. The stock solution was neutralized with 1 M NaOH (in ddH_2_O) to a pH of 7.5–8 and diluted in PBS, based on the weight of each mouse, directly prior to being administered. Mice were challenged 6 months after the last vaccination (after day 230) with 0.09 mg/kg of nicotine based on the molecular weight of free nicotine. For the IC challenge, mice were anaesthetized with isoflurane and 100 µL of the individually prepared nicotine solution was administered into the heart using a syringe and a 25 5/8” gauge needle. Five minutes after the IC injection, while still under anesthesia, the mice were euthanized. For the IN challenge, the mice were anaesthetized with isoflurane and 20 µL (10 µL/nare) was instilled drop wise ensuring full uptake of the nicotine dose. The mice were returned to the isoflurane chamber for 10 min and were then euthanized.

### 2.5. Euthanasia and Sample Collection

Mice were euthanized under excess isoflurane followed by a cardiac exsanguination and cutting of the diaphragm. All organs for each mouse were macroscopically examined post mortem for any abnormalities. Weights were recorded for the brain, lung, and heart before being stored on ice and processed for nicotine quantification. Sera was collected from the blood by centrifuging the nonheparinized blood tubes at 10,000 rpm for 5 min and stored at −20 °C or −80 °C until later analyses.

### 2.6. Organ Extraction and Lyophilization

All sera and organ samples were first acidified via the addition of formic acid (20 µL, LC/MS grade, Fisher Scientific, Fairlawn, NJ, USA) and frozen overnight at −80 °C before being lyophilized (Alpha 1-2 LD, CHRIST, Osterode am Harz, Germany).

Nicotine was extracted from lyophilized organ and sera samples using anhydrous ethanol (Commercial Alcohols, Brampton, ON, Canada) acidified with formic acid (0.1% *v*/*v*, LC/MS grade). Each sample was mixed with ethanol (400–800 µL depending on the physical size of the sample), homogenized using a pellet mixer (VWR International, Radnor, PA, USA), vigorously vortexed and left at 4 °C for 2 h before solids were pelleted by centrifugation at 10,000 rpm and 4 °C for 10 min. Supernatants (300–600 µL) were carefully removed and replaced with an equal volume of ethanol before being vigorously mixed and left at 4 °C for an additional 2 h. Solids were again pelleted by centrifugation and supernatants (300–600 µL) carefully removed. Combined supernatants were allowed to concentrate to dryness at room temperature. The remainders were reconstituted in ddH_2_O acidified with formic acid (0.1% *v*/*v*, LC/MS grade) and any solids pelleted by centrifugation at 10,000 rpm and 20 °C for 5 min. The amount of nicotine extracted was analyzed by RP-HPLC (10 µL injections, in duplicate) with detection by absorbance at 260 nm and quantification using a standard curve that was generated.

### 2.7. RP-HPLC for Nicotine Quantification

All analyses were performed using a Shimadzu Prominence series HPLC system (Shimadzu Corporation, Kyoto, Japan), equipped with a LC-20AB binary pump (Serial: L20124200883), SIL-20A HT autosampler (Serial: L20345256104), CTO-20AC temperature controlled column oven (Serial: L2021525077), SPD-M20A photodiode array detector and CBM-20A communications bus (Serial: L20235154327). All equipment was controlled by Shimadzu Lab Solutions Lite software version 5.71 SP2. For separation, an Ultra C18 column, 3 μm, 150 × 4.6 mm (RESTEK Corporation, Bellefonte, PA, USA) was used. Samples were analyzed at a constant solvent flow rate of 1.0 mL/min at 35 °C using a binary gradient (Table 1). Solvent A consisted of a ddH_2_O (0.2 μm filtered) and solvent B consisted of 20% ddH_2_O in acetonitrile (HPLC grade, Fisher Scientific, Fairlawn, NJ, USA) with each solvent containing 0.18% formic acid (*v*/*v*, LC/MS grade) and 0.15% triethylamine (*v*/*v*, HPLC grade, Fisher Scientific, Fairlawn, NJ, USA).

### 2.8. Bronchoalveolar Lavage (BAL)

Five weeks after the final vaccination, mice were euthanized as described above and post-mortem the trachea was cannulated with PE tubing attached to a 23 × 1 1/3” gauge needle. The lungs were slowly inflated with 0.5 mL PBS, which was slowly retracted and collected for each mouse. BALs were centrifuged at 3000 rpm for 5 min to pellet cells and the supernatants were collected and stored at −20 °C until later analyses.

### 2.9. Anti-Nicotine ELISAs

Sera (day 56 or after day 230) and BALs were analyzed via an indirect anti-nicotine ELISA as previously described [11]. Briefly, plates were coated with poly-lysine nicotine overnight at 4 °C before washing with TBST. Samples were diluted in TBST starting at 1:600 to >1:76,800 for sera, neat or 1:50 for the BAL and added to the plate to incubate for 1 h. Goat biotinylated anti-mouse IgG or IgA was diluted 1:10,000 in TBST and added to the plate for 1 h before adding streptadvidin alkaline phosphatase for another hour. para-Nitrophenylphosphate (pNPP) diluted in pNPP buffer was added, and the reaction stopped after 30 or 45 min with 3N NaOH. Results were read using a Synergy H4 Microplate Reader (BioTek, Winooski, VT, USA) at an absorbance of 405 nm with a subtraction of 490 nm.

## 3. Results

### 3.1. Physiological Conditions

**Figure 1:**
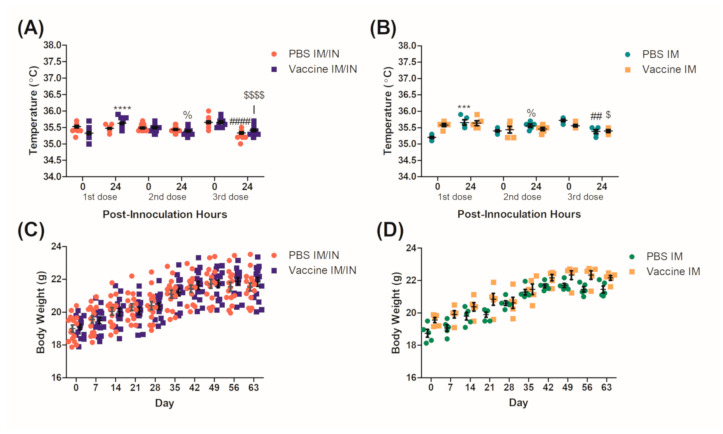
Clinical observations of BALB/c mice during the vaccination protocol. Female BALB/c mice had corporal temperatures recorded immediately before and 24 h after each (**A**) intramuscular (IM)/intranasal (IN) vaccination or (**B**) IM vaccination event. Mice were also weighed once a week starting on day 0 (first vaccination) until day 63 of the vaccination protocol for the (**C**) IM/IN vaccinated and the (**D**) IM vaccinated mice. Data for each figure are represented as ± SEM, (**A**) and (**C**) *n* = 15, (**B**,**D**) *n* = 5 for each group. Statistical analyses for (**A**) were determined by an unpaired two-tailed T-test, % *p* = 0.0255 and ****, ####, $$$$ *p* < 0.0001 as compared to the 24 h prior measurement for each dose, respectively. Statistical analyses for (**B**) was determined by an unpaired two-tailed T-test for % *p* = 0.0285, ## *p* = 0.012, *** *p* = 0.0007 as compared to the 24 h prior measurement for each dose/group respectively and a Mann–Whitney U-test for $ *p* = 0.0442 as compared to the 24 h prior measurement.

Mice were vaccinated using either the heterologous (IM/IN) or the homologous (IM) vaccination strategy and their weights and temperature were recorded to evaluate basic signs of toxicity that could be associated with the vaccine. Temperatures were recorded both before and 24 h after each vaccination to evaluate whether a fever was present. Although not clinically relevant, significant increases and decreases in temperature were seen across all groups for both the control and the vaccinated mice (Figure 1A,B). Mice were also weighed once a week to monitor their growth for any possible anorexia and associated weight loss that could be a result of vaccine-related toxicity. No significant decreases in weight were observed for any group or route of administration over the vaccination protocol (Figure 1C,D). Mice were observed daily, and no issues were recorded that were associated with the vaccine and all mice survived until the nicotine challenge.

### 3.2. Immunological Responses

**Figure 2:**
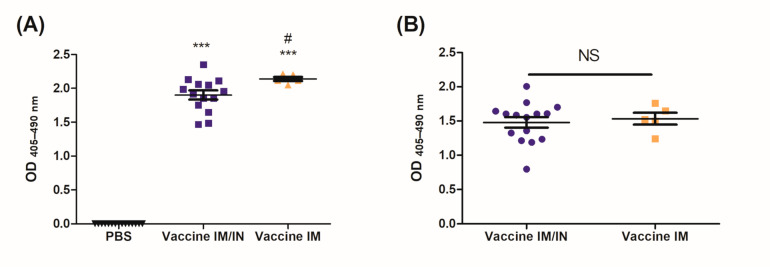
Levels of anti-nicotine IgG in the sera after the final vaccination. Mice were vaccinated on days 0, 21 and 42 using a control (PBS delivered IM/IN or IM), or the vaccine either delivered heterologously (IM/IN) or IM. Sera was collected on (**A**) day 56 or (**B**) more than 6 months (day 230) after the final vaccination, and analyzed using an indirect ELISA for total anti-nicotine IgG antibodies. For both (**A**,**B**), sera was diluted at 1:9600, *n* = 5–15 and data are represented as ± SEM. Statistical significance was determined by an ANOVA with a Tukey HSD, # *p* ≤ 0.05 as compared to Vaccine IM/IN, *** *p* ≤ 0.001 as compared to the PBS control. NS = not significant.

Systemic anti-nicotine antibodies were analyzed, with these representing sera levels 2 weeks (day 56) and 6 months (day 230) after the final vaccination to assess the stability of the antibodies. Significant levels of anti-nicotine IgG were present in the sera of mice vaccinated using either the IM/IN or IM strategy as compared to the PBS group. The IM group had significantly higher levels of anti-nicotine antibodies as compared to the IM/IN vaccinated mice after 56 days (Figure 2A). Six months after the final vaccination, elevated levels of anti-nicotine antibodies were still present in the sera of the mice vaccinated either IM/IN or IM. The IM vaccinated mice experienced a greater decrease in the amount of total anti-nicotine IgG in the sera as it was now on par and not significantly different from the IM/IN vaccinated mice (Figure 2B).

**Figure 3:**
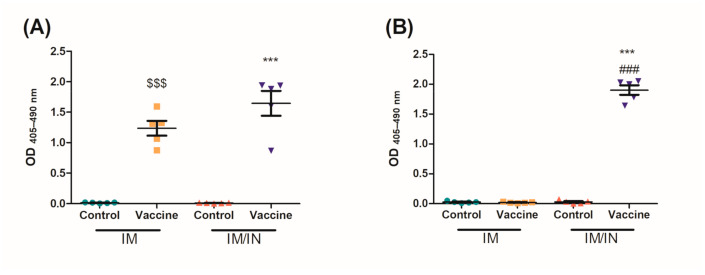
Levels of mucosal anti-nicotine antibodies in the lung after the final vaccination. Mice were vaccinated on days 0, 21 and 42 using PBS, or the vaccine either delivered heterologously (IM/IN) or IM. Five weeks after the final vaccination, bronchoalveolar lavages were assessed for anti-nicotine (**A**) IgG (1:50 dilution) and (**B**) IgA (neat) using an indirect ELISA. Data are represented as ± SEM and *n* = 5 for each group. Statistical significance was determined by an ANOVA with a Tukey HSD, $$$ *p* ≤ 0.001 and *** *p* ≤ 0.001 as compared to their respective controls, ### *p* ≤ 0.001 as compared to the IM vaccine group.

Mucosal antibodies are able to be induced through IN instillations of the vaccine. Five weeks after the final vaccination, BALs were collected to assess mucosal anti-nicotine IgA and IgG in the lung. Both the IM and IM/IN vaccinated mice had significant levels of anti-nicotine IgG present (Figure 3A). Although not significantly different, it is clear that the IM/IN vaccinated mice are trending to have higher levels of anti-nicotine IgG in the lung. The IM/IN vaccinated mice were the only group with significant levels of anti-nicotine IgA in the BAL, which was significantly higher as compared to the IM vaccinated group (Figure 3B).

### 3.3. Nicotine Challenge and Distribution

In vivo nicotine challenges remain the gold standard with which to establish whether a nicotine vaccine would be able to act as an effective anti-smoking therapeutic. This method measures the distribution of nicotine to various organs, including the brain and the blood, to establish whether the antibodies are able to block nicotine from going to the brain. We challenged both the IM and IM/IN vaccinated mice using a nontraditional IN instillation or IC injection with 0.09 mg/kg of nicotine, 6 months after the final vaccination.

**Figure 4:**
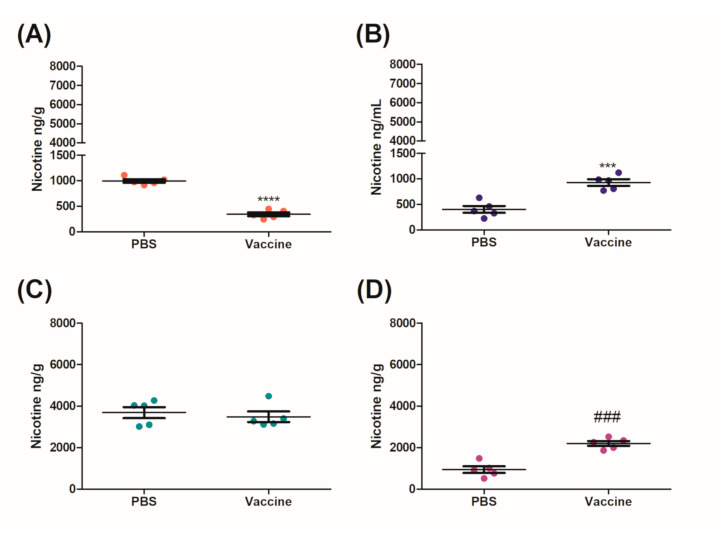
Distribution of nicotine in mice vaccinated IM after a mucosal nicotine challenge. IM vaccinated mice were challenged with 0.09 mg/kg of nicotine by an IN instillation of 10 µL per nare. After 10 min the mice were euthanized by a cardiac exsanguination and organs were collected for nicotine distribution analysis. (**A**) Brain, (**B**) sera, (**C**) lung, (**D**) heart and data are represented as ± SEM and *n* = 5 for each group. Statistical significances were determined by an unpaired two-tailed T-test, ### *p* = 0.0002, *** *p* = 0.0005 and **** *p* < 0.0001.

**Figure 5:**
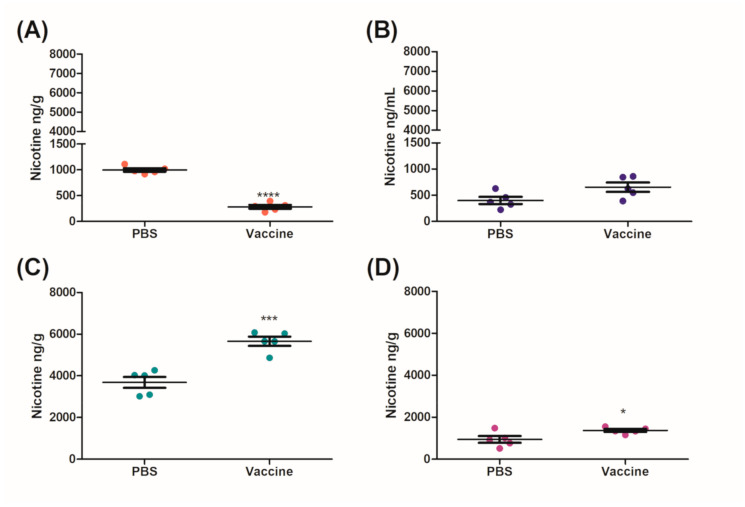
Distribution of nicotine in mice vaccinated IM/IN after a mucosal nicotine challenge. IM/IN vaccinated mice were challenged with 0.09 mg/kg of nicotine by an IN instillation of 10 µL per nare. After 10 min the mice were euthanized by a cardiac exsanguination and organs were collected for nicotine distribution analysis. (**A**) Brain, (**B**) sera, (**C**) lung, (**D**) heart and data are represented as ± SEM and *n* = 5 for each group. Statistical significances were determined by an unpaired two-tailed T-test, * *p* = 0.0384, *** *p* = 0.0004, and **** *p* < 0.0001.

**Figure 6:**
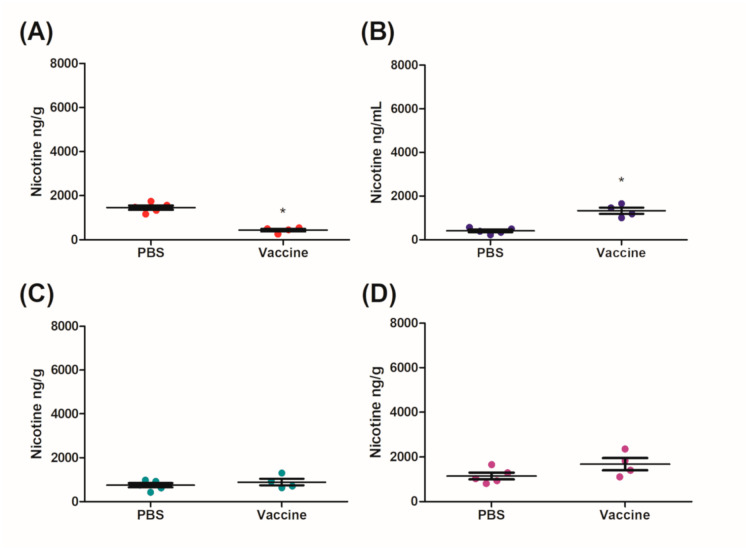
Distribution of nicotine in mice vaccinated IM/IN after a systemic nicotine challenge. IM/IN vaccinated mice were challenged with 0.09 mg/kg of nicotine by an IC injection of 100 µL. After 5 min the mice were euthanized by a cardiac exsanguination and organs were collected for nicotine distribution analysis. (**A**) Brain, (**B**) sera, (**C**) lung, (**D**) heart and data are represented as ± SEM and *n* = 4^–5 for each group. Statistical significances were determined by a Mann–Whitney U-test, * *p* = 0.0159. ^ One mouse was removed because of a technical issue during the intracardiac challenge.

For each of the challenge routes, the total amount of nicotine recovered from the brain, sera, lung, and heart was calculated from the organs as per HPLC/total amount of nicotine administered × 100%. The optimized nicotine detection method and analyses resulted in greater than 47% combined recovery of nicotine (Table 2). The IN challenge had the highest recovery of nicotine from blood, brain, heart, and lung at ≈56%, likely due to the increased levels of nicotine found in the lung after challenge for both the control and vaccinated mice (Table 2). The results from the HPLC analyses were normalized to the weights of the organs of each respective mouse and presented as either ng/g or ng/L in Figure 4, Figure 5 and Figure 6.

After 10 min, mice that were vaccinated IM had a significant 2.90-fold decrease in the amount of nicotine that was in the brain as compared to the control group (Figure 4A). Subsequently, this resulted in a significant increase in the levels of nicotine remaining in both the sera (2.41-fold) and the heart (2.34-fold) as compared to the control group (Figure 4B,D). There was no significant change in the amount of nicotine that was present in the lung (Figure 4C).

Mice vaccinated IM/IN were also challenged IN with 0.09 mg/kg nicotine after day 230 for 10 min. Similarly to the IM group, there was a significant decrease of nicotine in the brains of mice vaccinated IM/IN as compared to the control group (Figure 5A). The fold change between the brains of the control and the vaccinated mice was 3.54-fold (Figure 5A) which was higher than the 2.90-fold change for the IM vaccinated group (Figure 4A). The vaccinated mice had 1.45-fold and 1.54-fold increases of nicotine in the sera (Figure 5B) and the lung (Figure 5C), respectively. Although there was no significant difference in the amount of nicotine detected in the sera of the control and vaccinated group, there was a significant difference in the amount of nicotine detected in the lungs of the vaccinated mice as compared to the controls (Figure 5C). There was also a significant increase of nicotine in the heart as compared to the control (Figure 5D). Finally, an additional group of the IM/IN vaccinated mice were challenged using a traditional systemic method via an IC injection of nicotine at the same concentration of 0.09 mg/kg. The brains of the mice that were vaccinated IM/IN had a significant reduction in the amount of nicotine present as compared to the nonvaccinated controls (Figure 6A). This decrease resulted in approximately a 3.34-fold reduction of nicotine that was able to cross the blood–brain barrier (Figure 6A). There was also a significant 3.24-fold increase in the amount of nicotine that was detected in the sera (Figure 6B). The systemic method resulted in the least amount of nicotine in the lung (Figure 6C) and the heart (Figure 6D), with no significant differences between the vaccinated and unvaccinated groups.

## 4. Discussion

Nicotine vaccines continue to be sought after as a therapeutic alternative to help people quit smoking tobacco products. As previously reported, the vaccine formulation design is focused on mucosal immunity. The core of this conjugate nicotine vaccine platform is composed of a bacterial-derived adjuvant (BDA) from either *N. meningitides* or *V. cholerae* and both have been used as part of effective human vaccines [11,12,19,20,21,22]. The mucosal multiadjuvanted BDA is able to stimulate immune responses by activating antigen-presenting cells leading to effective adaptive immune responses [20], reviewed in [22]. The addition of a non-natural peptide serves as a matrix that facilitates nicotine conjugation and promotes humoral immune responses [20]. We have demonstrated that a heterologous IM/IN vaccine was immunologically superior to the homologous IM administration because of its ability to produce both systemic and mucosal antibodies [11].

In this study, using the same vaccination strategy as previously described, we monitored the health of the mice over the vaccination protocol and were able to demonstrate that significant levels of anti-nicotine antibodies were generated by either administration route. These anti-nicotine antibodies remained 6 months after the final vaccination and could still significantly reduce the amount of nicotine in the brain as compared to nonvaccinated controls.

Physiological conditions were observed and recorded during the vaccination protocol for indications of an adverse response to the BDA-conjugate nicotine vaccine. Mice in general are close to humans with respect to corporal temperature and on average are recorded as 36.6 °C ± 2.0 [23], which was consistent with our results. Although there were significant increases and decreases in temperature that occurred over a 24 h period, these would not be considered physiologically relevant as none of them varied more than 1 °C as compared to the baseline temperatures or would suggest a fever (Figure 1A,B). The weights of the mice were also recorded weekly as changes in weight could be attributed to toxicity associated with the vaccine, specifically the IN route where behavioral changes could result in reduced food consumption and anorexia [24]. No significant decreases in weights were recorded throughout the vaccination protocol with both the controls and the vaccinated groups (Figure 1C,D) steadily gaining weight on par with Charles River’s growth curves for the BALB/c strain [25]. We had previously demonstrated a more robust safety profile [11,19] and used the parameters mentioned to ensure the health of the mice throughout the protocol so that they were healthy for the nicotine challenge.

The BDA-conjugate nicotine vaccine induced significant levels of systemic and mucosal anti-nicotine IgG when administered either IM/IN or IM (Figure 2 and Figure 3A); additionally, the IM/IN vaccination strategy induced significant levels of mucosal IgA (Figure 3B). Our previous batch testing for the stability of the anti-nicotine antibodies induced by the mucosal administration provided evidence that the antibodies were stable in the lung for 8 months. We chose the challenge time point of 6 months based on the stability data from Figure S1A,B. The results from the nicotine distribution from Figure 5 would suggest that the mucosal antibodies, particularly the anti-nicotine IgA, are stable for 6 months after the final vaccination. Although the systemic antibodies were stable for 6 months using either route of administration, the IM vaccinated mice experienced a more noticeable drop in anti-nicotine antibodies over the same time frame, suggesting that the IM/IN vaccinated mice had a more stable antibody response and potentially better plasma B cells. The half-life of IgG in mice is approximately 6–8 days [26], and without plasma B cells, the levels of systemic antibodies would have decreased over time, eventually leading to an undetectable or clinically insignificant amount of antibodies directed against nicotine. Investigations to elucidate the mechanism of how the same conjugate-nicotine vaccine formulation is able to induce better responses when administered mucosally are in progress.

After 6 months, the IM and the IM/IN vaccinated mice were challenged by an IN instillation of 0.09 mg/kg based on free nicotine, or 0.257 mg/kg nicotine tartrate, which would be the equivalent of 16 cigarettes [27]. We had previously used a lower challenge dose [12], but with the optimized vaccination strategy, wanted to put additional pressure on the available antibodies by increasing the challenge dose. Despite appearing to be a large single dose of nicotine, it is more physiologically relevant as the average person who smokes tobacco has 17 cigarettes per day [27]. It was clear from the distribution results that the IM/IN vaccinated mice had higher fold reductions of nicotine in the brain, as compared to the IM vaccinated group when challenged IN. The reduction in the brain was 72% as compared to 65%, respectively (Figure 4A and Figure 5A). Since both the IM and IM/IN groups had similar levels of anti-nicotine IgG in the sera (Figure 3), we believe based on Figure S1B, this difference was attributed to the mucosal anti-nicotine IgA antibodies generated by the IM/IN route. These mucosal antibodies were able to keep a significant amount of nicotine in the lung as compared to the control group (Figure 5C). If the mucosal anti-nicotine IgG was able to noticeably capture nicotine in the lung, we would have recovered more nicotine in the lungs of the IM vaccinated mice as compared to the control (Figure 4C) due to the IgG leaking into the lungs. While the IM vaccinated mice had more nicotine recovered in the sera, this did not result in less nicotine in the brain as compared to the IM/IN vaccinated mice. It is clear that not only do the anti-nicotine antibodies in the lung reduce the burden on the systemic antibodies, but they also have increased binding capacity, resulting in a greater decrease of nicotine in the brain and potentially more therapeutically effective. This is not surprising as IgA exists in the mucosa most often as a dimer, but can also exist as a larger polymer [28] resulting in at least a 2-fold higher binding capacity per antibody as compared to IgG. The lung also acts as a physical barrier which slows down nicotine distribution [29], allowing the antibodies in the lung more time to interact with nicotine before it exits the lung, which would make mucosally administered anti-nicotine vaccines a more attractive approach.

The physiological barrier of the lung was also the reason for the difference in time between the IC and IN challenge. Our previous IC challenge using [^3^H]-nicotine [12] was 2 min and in this study it was extended to 5 min. Taking the 5–7 min half-life of nicotine in mice [27] into consideration and our longer IC challenge time, we used 10 min as our challenge time point for the IN administration.

IM/IN vaccinated mice were also challenged systemically with an IC injection using the same concentration of nicotine as the IN instillation. There was a stark contrast between the different challenge routes; specifically, there was less nicotine in the lungs of the mice challenged by IC injection as compared to the IN instillation. This inability of nicotine to interact with the lung in the IM/IN vaccinated mice resulted in the burden of the nicotine resting solely on the systemic antibodies. Comparatively, the mucosal and systemic antibodies seen in Figure 5 were able to capture nicotine at a greater capacity than the systemic antibodies alone as seen in Figure 6 with a 72% reduction as compared to 70%, respectively. However, both groups of IM/IN vaccinated mice demonstrated antibodies with superior neutralizing capabilities over the IM vaccinated mice (65%), regardless of the challenge route.

Current parenteral and mucosal nicotine vaccines have been published with varying degrees of efficacy [13,14,15,16,17,18,30,31,32] and they all demonstrate significant levels of anti-nicotine antibodies that are able to block nicotine from entering the brain. Despite this similarity, the challenge and nicotine analysis methods vary and nicotine recovery tends to be low. SC and IP administration of nicotine result in a slower absorption as compared to inhaled or IV administered nicotine [27,33] and are most commonly used for nicotine administration outside of addiction models [33]. The IN challenge represents a method that is less invasive, more physiologically relevant and less technically challenging as compared to an IC (IV), IP or SC challenge. The IN and IC challenges because of their rapid distribution and absorption could indirectly be more of a burden for the readily available antibodies and act as a more stringent method to assess our vaccine, as compared to IP or SC challenges. While, it is difficult to directly compare one study to another because of confounding factors including the amount of nicotine based on the weight of either free nicotine or nicotine tartrate, animal species, nicotine administration route, challenge time points, and analysis methods; it is evident that this new method, using lyophilization and RP-HPLC, has a 47–56% recovery and demonstrated the physiological differences between mucosal and systemic administration of nicotine in mice (Table 2). The remainder of the nicotine, based on the recovery time and quick half-life of nicotine in mice, could have been metabolized to a nicotine analogue, such as cotinine, which was not assessed by our method.

Nicotine rapidly distributes in the body and is able to reach the brain in 7 s [34] and when inhaled reaches peak accumulation in the brain after 3–5 min [34]. Due to this rapid distribution, it may not be possible to absolutely reduce the amount of nicotine in the brain. The continuous reduction of nicotine, a result of the nicotine–antibody complex, could lead to a decrease in nicotine dependence leading to smoking cessation [35]. The stability of the antibody–nicotine complex is important for the ability of the nicotine vaccine to act as an effective therapeutic. A strong complex will allow the nicotine to be removed by the immune system or slowly released from the complex without generating spikes of nicotine that fuel the addiction cycle [36]. This would rely on having anti-nicotine antibodies with high affinity and avidity. Based on both the IN and IC challenge methods, the antibody-nicotine complex is stable for at least 5–10 min, acting as a dynamic measure of avidity. This could allow for a slow release of nicotine over a longer time frame, reducing the need for a subsequent cigarette and helping as a therapeutic for smoking cessation.

Traditionally, vaccine efficacy has been demonstrated by the antibodies’ ability to recognize and neutralize the vaccine-specific antigen, and nicotine is no exception. However, nicotine vaccines have an additional hurdle to overcome, which is whether the vaccine can help to curb addictive behaviors. Nicotine self-administration models have been used to study behavior, most commonly of rats, when nicotine is administered as either short bursts or over an extended period of time [37]. While each model has contributed to the study of nicotine addiction, they may not represent the most appropriate models for evaluating a vaccine with respect to behaviors and addiction due to the high levels of nicotine and the exposure time. Future investigations could focus on whether the IM/IN nicotine vaccine is able to reduce the levels of systemic nicotine below levels associated with addiction and correlate these results with the concentration, stability and avidity of the anti-nicotine antibodies.

## 5. Conclusions

We demonstrated that a mucosal conjugate nicotine vaccine can induce stable antibody responses that are able to significantly block nicotine from reaching the brain 6 months after the final vaccination. The ability to neutralize nicotine depends on the route of challenge. It has been suggested that a minimum threshold of anti-nicotine antibodies is required in order to be an effective therapeutic [10]. However, our data suggests that the location, avidity and affinity may be important based on our distribution results in the lung, sera and brain and the ability of the antibody–nicotine complex to last up to 10 min. The novel extraction and analyses of nicotine lead to increased recovery of nicotine and may be more representative of the true neutralization capacity of the antibodies generated by the nicotine vaccine. Additionally, the IN instillation for the nicotine challenge represents a less invasive and more physiologically relevant strategy for demonstrating the ability of both the mucosal and systemic antibodies to interact with nicotine. This, in addition to our previous publications, further demonstrates the ability of the BDA-conjugate nicotine vaccine administered IM/IN to be a promising therapeutic for smoking cessation.

## Figures and Tables

**Table 1:** Solvent gradient program for the analysis of nicotine in vivo distribution using water (**A**) and 20% water in acetonitrile (**B**), both with 0.18% formic acid and 0.15% triethylamine (*v*/*v*).

Time (min)	Solvent	
	A (%)	B (%)
0	100	0
5	100	0
15	0	100
20	0	100
25	100	0
28	100	0

**Table 2:** The total average percentage of nicotine recovered from the brain, sera, lung, and heart of each challenge group ± SEM.

	IM/IN Route		IM Route Intranasal Challenge
	Intracardiac Challenge	Intranasal Challenge	
PBS	47.05 ± 3.55 (*n* = 5)	55.57 ± 3.46 (*n* = 5)	55.57 ± 3.46 (*n* = 5)
Vaccine	47.31 ± 0.80 (*n* = 4)	55.66 ± 1.88 (*n* = 5)	53.31 ± 1.76 (*n* = 5)

## Data Availability

Data requests related to this study can be sent to the corresponding author.

## Supplementary Materials

The following are available online at https://www.mdpi.com/2076-393X/9/2/118/s1, Figure S1: Levels of mucosal anti-nicotine IgA in the lung.
