# Supplementary material for: Assessing Neutralized Nicotine Distribution Using Mice Vaccinated with the Mucosal Conjugate Nicotine Vaccine
Source: Vaccines (Basel). 2021 Feb 3;9(2):118. doi: 10.3390/vaccines9020118 (PMC7913222; doi:10.3390/vaccines9020118)
Supplement: Supplementary file 1 [file vaccines-09-00118-s001.pdf]

Supplemental Figure S1

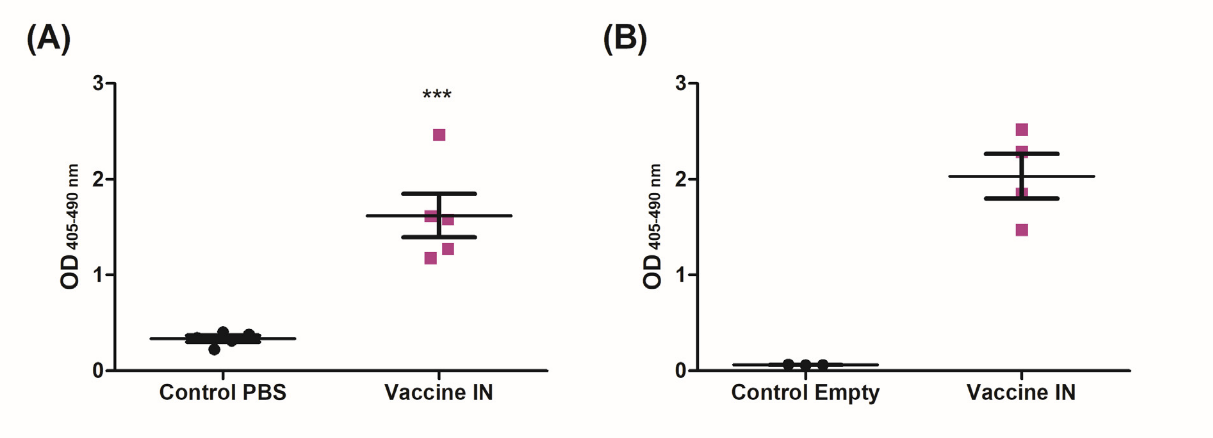

Figure S1: Levels of mucosal anti-nicotine IgA in the lung. Mice were vaccinated using the homologous intranasal vaccination strategy in two separate investigations. Mice were euthanized at the end of their respective trials and BALs were collected. Levels of anti-nicotine IgA were assessed by an indirect ELISA. A) BDA Trial 4_2015-16 nine weeks after the final vaccination, BAL diluted 1:2, ± SEM n=5. Statistical analysis was performed by an unpaired 2-tailed T test, ***p=0.0005. B) BDA Trial 1_2013-14 eight months after the final vaccination, no dilution of BAL, ± SEM, n=3 for the control and n=4 for the vaccine group. Control Empty is the BDA without nicotine.
